# Molecular Comparison of Bacterial Communities on Peripheral Intravenous Catheters and Matched Skin Swabs

**DOI:** 10.1371/journal.pone.0146354

**Published:** 2016-01-05

**Authors:** Md Abu Choudhury, Nicole Marsh, Shahera Banu, David L. Paterson, Claire M. Rickard, David J. McMillan

**Affiliations:** 1 NHMRC Centre of Research Excellence in Nursing (NCREN), Menzies Health Institute Queensland, Griffith University, Brisbane, Australia; 2 Alliance for Vascular Access Teaching and Research, Griffith University, Brisbane, Australia; 3 Inflammation and Healing Research Cluster, School of Health and Sports Sciences, University of the Sunshine Coast, Sippy Downs, Brisbane, Australia; 4 Royal Brisbane and Women’s Hospital, Brisbane, Australia; 5 Institute of Health and Biomedical Innovation, Queensland University of Technology, Brisbane, Australia; 6 University of Queensland Centre for Clinical Research, Royal Brisbane and Women's Hospital Campus, Brisbane, Australia; ContraFect Corporation, UNITED STATES

## Abstract

Skin bacteria at peripheral intravenous catheter (PIVC) insertion sites pose a serious risk of microbial migration and subsequent colonisation of PIVCs, and the development of catheter related bloodstream infections (CRBSIs). Common skin bacteria are often associated with CRBSIs, therefore the bacterial communities at PIVC skin sites are likely to have major implications for PIVC colonisation. This study aimed to determine the bacterial community structures on skin at PIVC insertion sites and to compare the diversity with associated PIVCs. A total of 10 PIVC skin site swabs and matching PIVC tips were collected by a research nurse from 10 hospitalised medical/surgical patients at catheter removal. All swabs and PIVCs underwent traditional culture and high-throughput sequencing. The bacterial communities on PIVC skin swabs and matching PIVCs were diverse and significantly associated (correlation coefficient = 0.7, *p*<0.001). *Methylobacterium* spp. was the dominant genus in all PIVC tip samples, but not so for skin swabs. Sixty-one percent of all reads from the PIVC tips and 36% of all reads from the skin swabs belonged to this genus. *Staphylococcus* spp., (26%), *Pseudomonas* spp., (10%) and *Acinetobacter* spp. (10%) were detected from skin swabs but not from PIVC tips. Most skin associated bacteria commonly associated with CRBSIs were observed on skin sites, but not on PIVCs. Diverse bacterial communities were observed at skin sites despite skin decolonization at PIVC insertion. The positive association of skin and PIVC tip communities provides further evidence that skin is a major source of PIVC colonisation via bacterial migration but microbes present may be different to those traditionally identified via culture methods. The results provide new insights into the colonisation of catheters and potential pathogenesis of bacteria associated with CRBSI, and may assist in developing new strategies designed to reduce the risk of CRBSI.

## Introduction

The insertion of a vascular catheter is a major risk factor associated with nosocomial infection [1]. In particular, peripheral intravenous catheters (PIVCs) are linked to catheter related blood stream infections (CRBSIs), with up to five percent of all bacteraemias being associated with PIVC use [2]. CRBSIs increase a patient’s risk of death, length of hospital stay, and are a significant cost to the health care system [3]. The predominant bacterial species associated with CRBSIs are *Staphylococcus aureus*, *S*. *epidermidis*, *Pseudomonas aeruginosa*, *Acinetobacter* spp and *Bacillus* spp [1, 4]. In most cases, bacteria causing CRBSIs are derived from the patient’s own skin or from health care staff [5, 6]. When present on the skin, these bacteria do not normally cause serious disease. However when the skin is breached, such as by the insertion of a PIVC, they can traverse this barrier and establish a systemic infection [1, 6]. Furthermore patients in hospital may also be immunocompromised, inhibiting their capacity to combat such infections [7].

Current hospital laboratory practice for diagnosis of pathogens causing CRBSI is based on PIVC tip investigation using the culture dependent Maki method [8]. While these methods are efficient in the identification of known major pathogens, they may underestimate overall microbial diversity due to microbial preference for agar media, microbial competition or the presence of non-culturable bacteria [5, 9–12]. Culture independent molecular studies have vastly expanded our understanding of bacterial diversity present in many environments, and are revealing the true diversity of bacteria present on the skin [9, 13]. Such studies have identified new human pathogens, and may also help to identify potential association between microbial communities and disease, as well as associations with body defences [9, 14, 15]. With respect to vascular catheters, several culture-independent studies have also demonstrated that complex bacterial communities, including common skin bacteria, are present on virtually all catheters even in patients without signs of infections [16–18].

To reduce the risk of CRBSI, catheter pre-insertion sites are decontaminated with antiseptics such as chlorhexidine gluconate in alcohol [19], and then covered with a dressing for up to 7 days. Here we have used culture-independent methods to analyse the bacterial communities present on catheters with that of matched skin samples taken from the insertion site both collected at removal. Knowledge of the bacterial load and diversity on the skin may be an indicator of the likely microbial community colonising PIVCs and the risk of CRBSI.

## Materials and Methods

### Collection and processing of samples

PIVCs and matching skin swabs were recovered from 10 adult patients participating in a larger randomised clinical trial of dressings. Catheters were inserted as part of normal clinical treatment in medical and surgical wards of the Royal Brisbane and Women’s Hospital in Queensland, Australia, a major tertiary-referral, teaching hospital. Research Nurses (ReN) screened patients for eligibility. Patients aged 18 years or more with a PIVC expected to be used for a minimum of 24 hours were recruited for the study. PIVCs were either in the forearm or wrist secured with four different dressings (SPU, standard polyurethane; BPU, bordered polyurethane; SSD, sutureless securement device; Glue, tissue adhesive) (Table 1). Exclusion criteria were that the patient had an existing BSI, was non-English speaking without an interpreter, was extremely diaphoretic, had burned or diseased skin at the PIVC site, or had existing skin tears or papery skin. Data were collected for each patient: age, sex, PIVC location, antimicrobial use, dwell-time, reason for removal, CRBSI diagnosis and blood culture reports.

**Table 1:** Patient demographics and clinical characteristics.

Patient number	Age	Sex	Duration of catheter in situ (hours)	Dressings securement method	Device location	Diagnosis	IV therapy (antibiotic usage)	Phlebitis
1	60	Male	139	SSD	Lower anterior left forearm	Surgical elective vascular	Cefazolin	No
2	19	Female	25	Glue	Right hand	Surgical elective gastrointestinal	Metronidazole and Ampicillin	No
3	62	Male	73	Glue	Posterior left wrist	Medical	Ceftozidine and Metronidazole	Yes
4	93	Female	92	Glue	Upper anterior right forearm	Medical	Ampicillin and Ceftriaxone	No
5	34	Male	46	SSD	Posterior lower right forearm	Medical	Dexamethasone and Rifampicin	No
6	63	Male	141	SPU	Lower anterior right forearm	Surgical elective thoracic	No	Yes
7	33	Female	192	SPU	Posterior right wrist	Surgical elective gynaecology	Metronidazole and Ceftriaxone	No
8	19	Female	28	SPU	Posterior left wrist	Medical	Flucloxacillin	Yes
9	44	Female	24	BPU	Posterior right wrist	Medical	Piperacillin and Tazobactam	Yes
10	53	Female	24	BPU	Posterior lower right forearm	Medical	No	ND

SPU, standard polyurethane; BPU, bordered polyurethane; SSD, sutureless securement device; Glue, tissue adhesive; ND, Not done

Prior to PIVC insertion, the skin was decontaminated with 2% chlorhexidine gluconate in 70% alcohol. Catheters were BD Insyte Autogard shielded IV catheters (Becton Dickinson, Utah, USA) inserted into the forearm or wrist. Maintenance of the catheter and dressings followed standard hospital protocols [20]. The skin samples from catheter insertion sites were swabbed by the ReN using sterile cotton swabs moistened with 200μl 0.9% sterile sodium chloride solution (Pfizer, USA). The PIVC skin insertion site (2 cm surface area) was swabbed for 10 seconds back, forward and rolling over onto the skin after removal of the dressing and just prior to PIVC removal. After PIVC removal, 1–2 cm from the distal end of the PIVC was detached with sterile scissors, and placed in a sterile container. Skin and PIVC tip specimens were transferred immediately to the microbiology research laboratory and stored at -20°C. The skin swabs were thawed on ice and vortexed in 1ml of PBS to dislodge bacteria, centrifuged and then resuspended in 200μl of PBS. One hundred microliters of this suspension was plated onto Horse Blood Agar (HBA; bioMerieux, Australia). PIVC tips were placed in 1.5ml microcentrifuge tubes containing 1ml PBS, briefly vortexed, and then 100μl of the bacterial suspension was plated onto HBA [8, 21]. The plates were then incubated at 37°C for 72 hours and monitored daily for bacterial growth. As controls, three unused sterile swabs and three unused sterile PIVCs were treated as described above.

### DNA extraction and Polymerase Chain Reaction (PCR)

PIVC tips, including PIVC controls (unused sterile PIVCs) in 1.5ml microcentrifuge tubes containing 1ml PBS were briefly sonicated and vortexed [17]. After removal of the PIVCs, the bacterial suspension was re-centrifuged and pellet suspended in 100μl PBS. The pellets from skin swabs including swab controls were resuspended in 100μl of PBS. Chromosomal DNA was then extracted from both the skin and PIVC tips using the ultraclean microbial DNA isolation kit (MO BIO Laboratories, Inc). To confirm the presence of bacterial DNA, 16S rRNA genes were amplified from each sample using the forward 27F (5/AGAGTTTGATCMTGGCTCAG3/) and 519R (5/GWATTACCGCGGCKGCTG3/) primers [22] utilising the GoTaq^®^ Green Master Mix (Promega, USA). PCR was performed using the following cycling conditions; 94°C for 4 minutes for one cycle; 94°C for 30 seconds, 56°C for 45 second and 72°C for 1 minute for 35 cycles; 72°C for 10 minutes. The resulting PCR products were visualised using agarose gel electrophoresis. DNA extraction contained measurable quantities of microbial DNA (>10ng/μl) from all samples and amplified with visible bands. On the other hand, negative controls, including each three unused PICV and swab controls, and PCR negative controls (water) did not posses quantifiable DNA and visible band after amplification.

### Microbial community analysis

Culture independent microbial community analysis was performed by the Australian Genome Research Facility Ltd, Brisbane, Australia. PCR amplicons were generated with the cycling conditions of 95°C for 7 minutes for one cycle and then 94°C for 45 seconds, 56°C for 1 minute and 72°C for 1 minute run up to 29 cycles and 72°C for 7 minutes for final extension, using AmpliTaq Gold 360 Mastermix (Life Technologies, Australia) for the primary PCR. A secondary PCR to index the amplicons was performed with TaKaRa Taq DNA Polymerase (Clontech). The resulting amplicons were measured by fluorometry (Invitrogen Picogreen). The eqimolar pool was then measured by qPCR (KAPA) followed by sequencing on the Illumina MiSeq with 2x300bp Paired End Chemistry. Raw sequencing read data has been submitted to and archived in The NCBI Sequence Read Archive (accession number SRP065095).

Paired-ends reads were assembled by aligning the forward and reverse reads using PEAR1 (version 0.9.5). Primers were trimmed using Seqtk (version 1.0). Trimmed sequences were processed using Quantitative Insights into Microbial Ecology (QIIME 1.8)4 USEARCH 2,3 (version 7.1.1090) and UPARSE software. Sequences were quality filtered using usearch tools. Full length duplicate sequences were removed and remaining sequences sorted by abundance. Singletons or unique reads in the data set were discarded. To obtain number of reads in each OTU, reads were mapped back to OTUs with a minimum identity of 97%. Taxonomy was assigned using Greengenes database5 (Version 13_8, Aug 2013) as implemented in QUIME.

### Statistical analysis

Statistical analysis was performed with IBM SPSS statistics version 21(IBM Corporation) and GraphPad Prism software package (GraphPad Software Inc.). Pearson correlation test was performed to determine the correlation of bacterial community structures and frequency between PIVCs and PIVC skin insertion sites [23, 24]. ANOVA was used to examine difference of relative abundance between phyla and genus present on PIVC tips and skin swabs. Unpaired t test was used to determine the difference in diversity present on PIVC tips and skin swabs. P values lower than 0.05 were considered statistically significant.

### Ethics

The study was approved by the Royal Brisbane and Women’s Hospital and Griffith University human research ethics committees (HREC/11/QRCH/152). All participants underwent informed consent, with written consent being obtained prior to enrolment. All patient identifiers were removed from samples with a unique study number assigned before they were used for research purposes.

## Results

### Patients and clinical characteristics

A total of 10 adult patients were included in this study, where 60% were females and the median age was 48 years. All PIVCs were short-term, varied between 1 to 7 days (average 3 days). Fifty percent of patients had post-removal phlebitis. Eighty percent of patients were receiving systematic antibiotic therapy during the observation period and stayed in the hospital for an average 11 days (Table 1). None of the PIVCs and associated skin samples was positive in bacterial growth on culture.

### Overview of bacterial diversity on PIVCs and associated skin sites

A combined total of 2,415,480 raw sequences were acquired from the PIVC tips and skin swabs. After quality filtering and chimera checking, 1,463,456 working sequences (358,205 from PIVC tips and 1,105,251 from skin swabs) with an average read length of 300 base pairs were obtained.

**Fig 1:**
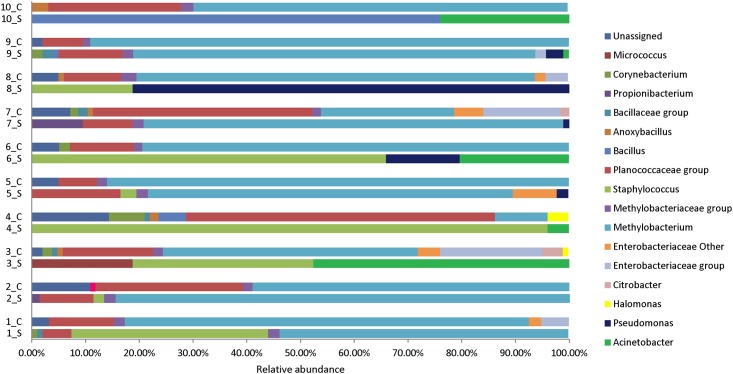
Bacterial Genus/Family detected on peripheral intravenous catheter (PIVC) tips and associated skin swabs at PIVC insertion sites. C represents PIVC tip and S represents skin swab.

**Fig 2:**
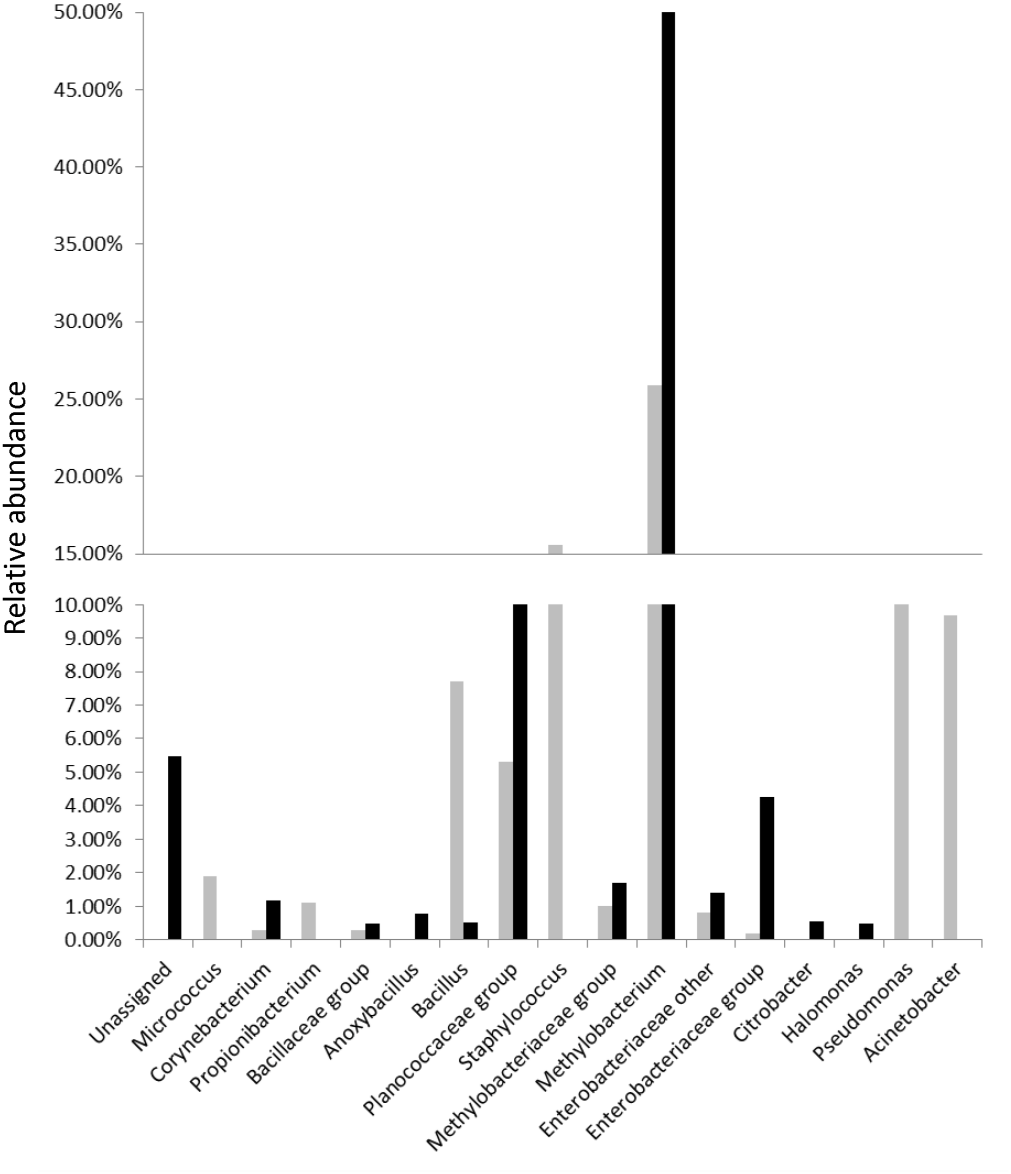
Proportion of Bacterial Genus/Family detected on all peripheral intravenous catheter (PIVC) tips and associated skin swab at PIVC insertion sites. PIVC tip and skin swab are represented by black and gray bar respectively.

Four bacterial phyla and sixteen genera were represented in the combined dataset. *Proteobacteria* (64% of reads) was the dominant phylum, followed by *Firmicutes* (31%), *Actinobacteria* (2%) and *Cyanobacteria* (1%). The abundance of sequences detected for each phylum in the PIVC tips and skin swabs were significantly different (*p*<0.05, two-way ANOVA). A side-by-side comparison of bacterial Genus/Family detected on PIVC tips and associated skin swabs at PIVC catheter sites is shown in Fig 1. The majority of sequences were classified as belonging to *Methylobacterium* (49%), *Staphylococcus* (13%), *Planococcaceae* group (13%), *Pseudomonas* (5%), *Acinetobacter* (5%), *Bacillus* (4%), *Enterobacteriaceae* group (3%), *Propionibacterium* (1%), *Corynebacterium* (1%) and *Micrococcus* genera (1%) (Fig 1). The average diversity (Simpson's index) at this level for PIVC tips and skin swabs was 0.60 and 0.78 respectively. This difference was not statistically significant (unpaired t test, *p*>0.05). *Methylobacterium* was the dominant genus in both PIVC and skin swab samples (Fig 2). Sixty-one percent of all reads from the PIVC tips and 36% of all reads from the skin swabs belonged to this genus. *Staphylococcus* was detected in 26% of all reads from the skin swabs. *S*. *aureus* was identified in skin swabs of two patients (Fig 2). On one patient’s skin, 96% of the sequences represented this genus. Sequences representing *Acinetobacter* (10%) and *Pseudomonas* (10%) were both observed in five skin samples. None of the PIVC tips analysed in this study were positive for sequences representing *Staphylococcus*, *Pseudomonas* or *Acinetobacter*. *Bacillus* was present on two skin swabs (average 39%) and one PVC tip (5%). Finally, the presence of these bacterial groups did not display any association with any of the clinical parameters including dressing types (*p*>0.05, two-way ANOVA).

### Relationship of diversity profile on PIVC tips and associated skin sites

**Fig 3:**
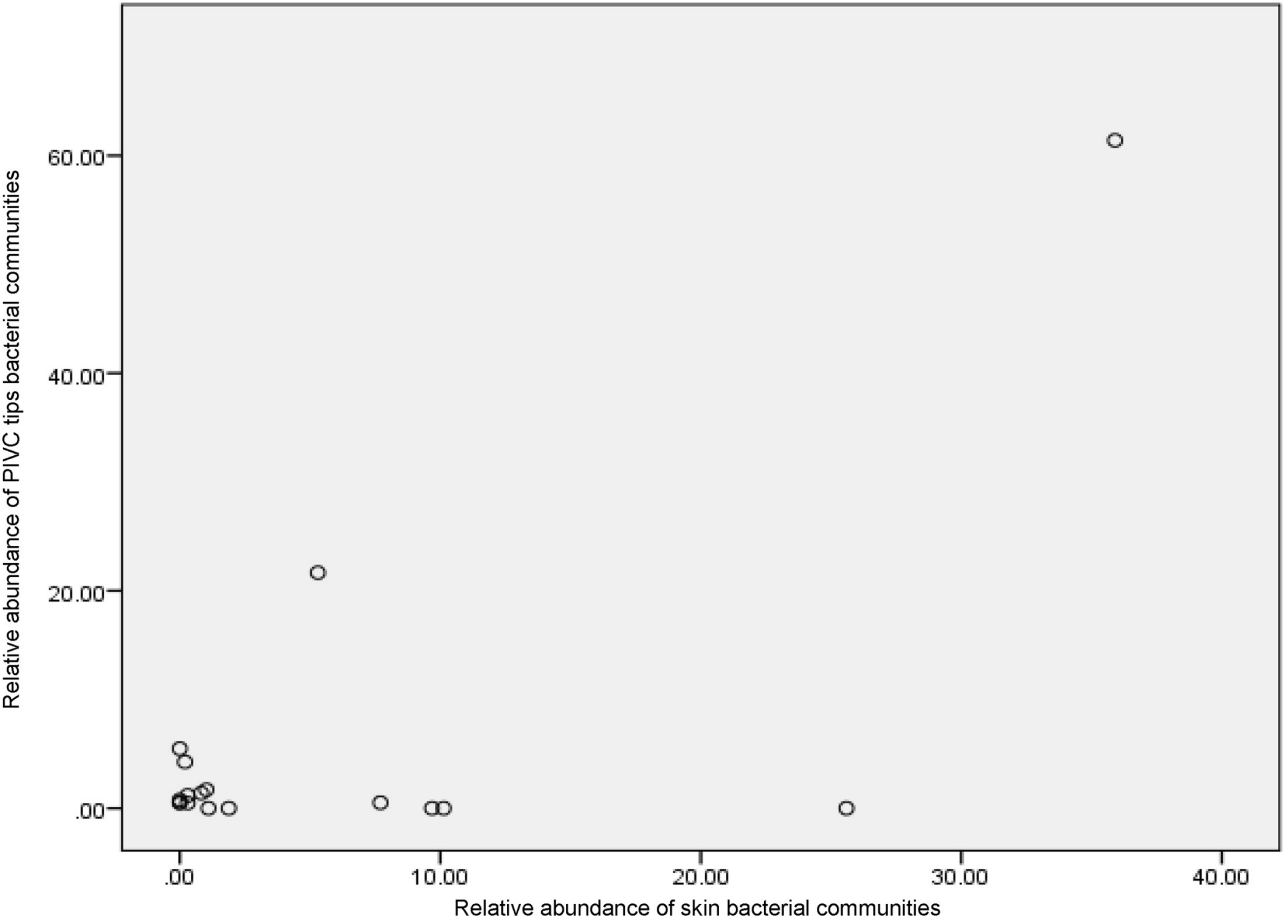
Relationship of the bacterial communities between PIVC tips and skin swabs at PIVC insertion sites. Pearson correlation showing the association of bacterial communities between PIVC tips and skin swabs at PIVC catheter sites were highly significant (correlation coefficient = 0.7; *p*<0.001).

The skin of the patient is reported to be the major source of pathogens causing CRBSI [6]. To determine if there was a correlation between all bacteria present on the skin, and bacteria recovered from PIVC tips, the distribution and abundance of bacterial genera present at these sites was compared for individual patients. A statistically significant correlation was observed in the distribution and relative abundance of bacterial genera from PIVC tips and skin swabs (correlation coefficient = 0.7; *p*<0.001). The most common sequences in both PIVC tip and skin swabs bacterial communities were *Methylobacterium*, *Planococcaceae* group and *Bacillaceae* group with an average number of sequences for each of these genera to be 61%, 22% and 1% consecutively in PIVC tips, and 35%, 5% and 8% consecutively in PIVCs skin sites (Fig 3). Here, *Planococcaceae* and *Bacillaceae* groups could not break down into genus level. The relative abundance of the most common genera in PIVC tips and skin swabs were not statistically different (*p*>0.05, two-way ANOVA). We also determined whether the bacterial genera were exclusively detected on either PIVC tips or skin swabs (Table 2). *Staphylococcus* (26%), *Pseudomonas* (10%), *Acinetobacter* (10%) and *Micrococcus* (2%) were detected only on skin swabs but not detected in PIVC tip communities. Furthermore, *Anoxybacillus* (1%), *Enterobacteriaceae* (4%), *Citrobacter* (1%) and *Halomonas* (1%), were detected only on PIVC tips but not detected in skin swab communities (Table 2).

**Table 2:** Bacterial genera/family detected on PIVCs and associated PIVC skin sites. Each genera/family presented with an average percentage of sequences from either PIVC or PIVC skin sites, or in both samples.

Both PIVCs and matched PIVC skin sites (%)	Only on PIVC skin sites not on PIVCs (%)	Only on PIVCs not on PIVC skin sites (%)
*Corynebacterium* (1%)	*Micrococcus* (2%)	*Anoxybacillus* (1%)
*Propionibacterium* (1%)	*Staphylococcus* (26%)	*Enterobacteriaceae* group (4%)
*Bacillaceae* group (1%)	*Pseudomonas* (10%)	*Citrobacter* (1%)
*Planococcaceae* group (14%)	*Acinetobacter* (10%)	*Halomonas* (1%)
*Methylobacterium* (46%)		
*Enterobacteriaceae* other (2%)		

## Discussion

Human skin harbors a huge diversity of bacterial communities, and serves as the major source of bacterial pathogens that contribute to CRBSIs [9, 13, 25, 26]. This is the first study using high-throughput 16S rRNA gene based pyrosequencing to directly compare bacterial communities on catheters with those of the surrounding skin. Our data show that the composition of bacterial communities present on PIVC tips correlates with the microbial community present at catheter insertion sites. Thus in addition to known pathogens on PIVC tips, catheters also become colonised with traditionally non-pathogenic bacteria present on the skin. The role that these organisms actually play in CRBSI is unknown. While it is unlikely that they contribute directly to pathogenesis, they may aid in colonisation, or conversely compete for colonisation in medical devices with known pathogens [5, 27, 28]. Both altruism and competition have been reported in biofilm communities [28–30]. Further to this, a protein expressed by *S*. *epidermidis* also induces host antimicrobial peptide (AMP) expression that inhibits the growth of group A *Streptococcus* and *S*. *aureus* in a murine model [15].

The overall bacterial diversity observed in this study was less than that reported in other studies [17, 31]. The samples in the study were taken from patients under clinical care, including use of 2% chlorhexidine gluconate in alcohol, to decontaminate skin sites prior to PIVC insertion. Chlorhexidine is an antiseptic that disrupts the bacterial cell wall, affecting osmotic equilibrium, and ultimately resulting in bacterial death [32]. Its use results in reduction in viable bacterial numbers at the site of insertion with ongoing effect over several days [33], and provides a strong rationale for the reduced diversity in these samples when compared to other studies [31]. Nevertheless the recovery of DNA from both skin and PIVC tip, with a different bacterial composition to that observed in other studies, suggests that some viable bacteria remained after treatment, or that PIVC insertion site was recolonised after some point after catheter insertion. Viable bacteria may remain in hair follicles and the lower dermis and thus may be shielded from antiseptics [34]. In our case, the culture of bacteria from skin at PIVC insertion sites indicates that some viable organisms were present at the insertion site at time of catheter removal. However, chlorhexidine is not equally effective for all classes of bacteria. For example, it is only moderately effective for Gram-negative bacteria [35]. *Methylobacterium*, the major bacterial genera detected in the study is Gram-negative. To our knowledge the chlorhexidine minimum inhibitory concentration (MIC) for *Methylbacterium* is unknown, and would be worthy of future research investigation.

*Methylobacterium* spp., was the dominant genera on both the PIVC tips and skin swabs. This contrasts to other studies which have reported *Stenotrophomonas* spp., as the major species recovered from PIVCs [17], and *Staphylococcus* spp. and *Corynebacterium* spp., as the major species recovered from skin [13]. Although *Methylobacterium* species are rarely thought to cause human infection [35–37], a catheter related infection associated with this species has recently been reported [37]. They have also been recovered from putatively sterile sites such as blood, bone marrow, sputum, pleural effusion, and skin [35, 38] and may be considered an opportunistic pathogen. *Methylobacterium* spp., are generally slow growing, exhibit resistance to disinfectants such as glutaraldehyde [36], chlorination [39] and dehydration, and form biofilms, particularly in settings in which water or liquid are present. Together these properties may help to explain its relative abundance after antiseptic treatment of catheter insertion sites. More broadly, *Methylobacterium* spp., have been reported as a pioneering biofilm species in non-medical settings in which antimicrobial coatings are used [10]. In these reports it was suggested colonisation of antibacterial surfaces with *Methylobacterium* provides a layer of cells to which more susceptible cells can attach. As many vascular catheters also possess antimicrobial surfaces (although not in this study) it is easily conceivable that these bacteria could play a similar role in this environment.

Although sequences for the known CRBSI pathogens, such *Staphylococcus* spp., *Pseudomonas* spp., and *Acinetobacter* spp., were observed in the PIVC skin samples, they were not present on the PIVC tips. No viable bacteria from isolates of these species were recovered in the study. Bacterial suspension from PIVC tips was plated onto HBA after vortexing in an attempt to identify viable bacteria on the intra and extra -luminal surfaces of the catheter. The initial vortexing step was designed to dislodge bacteria on both catheter surfaces, a technique that was described in clinical guidelines [40], and by others [8, 21, 41] for the identification of viable bacteria present on both intra and extra-luminal surfaces. Finally, only 20 samples from 10 patients were included in this study. Previous studies that used 15–30 PICV samples detected both quantitative and qualitative differences of bacterial community [16, 17]. Given the large number of bacterial species present of the skin, and variation in populations, a larger study may provide more detailed resolution of bacterial composition on the PIVC tip and associated skin swab samples. Moreover these studies may provide valuable data on the effect of different clinical interventions, such as use of different antibiotics, on bacterial population structures.

These results support current decontamination practices as adequate in reducing the risk of CRBSI. Chlorhexidine and other antiseptics are a critical and important part of infection control practice. However, reduced susceptibility to chlorhexidine in *S*. *aureus*, *S*. *epidermidis* and other major nosocomial pathogens has been reported [42, 43]. Similar to antibiotics, ongoing use of antiseptics such as chlorhexidine creates an environment in which increased tolerance may select for bacteria with increased tolerance or resistant. Therefore, it is possible that inadequate skin antisepsis could play a part in bacterial colonisation on PIVC skin sites and subsequent migration onto the PIVC tips.

## Conclusions

This study provides new insights into the colonisation of catheters and potential pathogenesis of bacteria associated with CRBSI, and may assist in developing new strategies designed to reduce the risk of CRBSI. Bacterial profiles on skin at catheter entry sites have major implications for bacteria that colonise catheters and their potential produce CRBSIs. Next-generation sequencing (NGS) provides a robust tool for characterising these communities. Importantly, these approaches can reveal, and capture, the genetic potential present in complex microbial communities without isolating the microorganisms.

A better understanding of bacterial community structures on PIVC tips and associated skin swabs may result in a new model for measuring and/or predicting PIVC tip colonisation and associated CRBSIs. However, to our knowledge no previously reported studies have determined the bacterial diversity on skin at catheter entry sites and their potential association with catheter bacteria by NGS techniques.
